# Pubic Arch Angle Postural Mobility Evaluation in Different Patient's Positions for Clinical Research

**DOI:** 10.7759/cureus.75898

**Published:** 2024-12-17

**Authors:** Marco Siccardi, Alessia Selmin, Cristina Valle

**Affiliations:** 1 Obstetrics and Gynecology, ApsDEHA, Savona, ITA; 2 Obstetrics, University of Padua, Padua, ITA; 3 Yoga and Cranial Osteopathy, ApsDEHA, Savona, ITA

**Keywords:** childbirth, mobility limitation, obstetrics, pelvimetry, posture, pubic arch angle, ultrasound

## Abstract

Childbirth is a dynamic process involving mutual adaptation between the maternal pelvis and the presenting fetal part. The ability of the pelvis to maintain optimal mobility during labor plays a crucial role in achieving favorable obstetric outcomes. The pubic arch angle (PAA) increases amplitude during pregnancy, showing pelvic tissue adjustment. The PAA evaluated with ultrasound in a single position predicts the risk of dystocia in labor and, consequently, anal sphincter trauma and incontinence after delivery. The hip flexion degree was found to reduce lumbar lordosis, shift the sacral promontory, affect the pubic arch angle, and increase pelvic diameter, creating more space for the fetus to descend during labor. Studies with magnetic resonance have demonstrated the modification of pelvic diameters and the PAA with maternal position change in the degree of hip joint flexion.

The present technical report intends to describe the technique for evaluating the PAA amplitude change in supine, kneeling, and standing patients' different leg positions. The procedure is designed for clinical research in labor biomechanics. The supine leg positions for pubic angle measurement can vary from hyperextension, as in Walcher's position, to neutral supine position, mild hip flexion, and hyperflexion, which is the position of the McRoberts maneuver. The kneeling and standing positions mimic labor and delivery in the flexible sacrum maternal positions. The 2D ultrasound technique can assess the PAA in the clinical research setting during the obstetrical examination. The transducer transversely positioned on the perineum shows the pubic symphysis and the two symmetrical ischiopubic branches, as described in the literature.

Evidence from ultrasound, magnetic resonance imaging, and computational modeling highlights the adaptability of pelvic structures influenced by hip flexion and soft tissue elasticity. Preliminary studies confirm significant positional differences in pubic arch angle and pelvic measurements, supporting the clinical relevance of assessing pelvic mobility.

The proposed ultrasound-based approach for evaluating PAA measurements in various maternal positions offers a practical tool for research in labor management and predicting vaginal birth outcomes. Ongoing research aims to elucidate further the relationship between pelvic dimensions in different maternal positions, fetal progression, and obstetric outcomes, contributing to safer, more effective childbirth practices.

## Introduction

Pregnancy and childbirth are appropriately described as dynamic processes [1]. The term emphasizes that these events involve continuous changes, adjustments, and interactions between the mother's body and the developing fetus throughout pregnancy and labor. A mutual adaptation between the maternal pelvic bone and soft tissue and the presenting fetal part highlights the reciprocal mobility to accommodate the fetus and facilitate the birthing process [1]. The maternal-fetal disproportion nosology refers to the reciprocal size of the birth canal space and fetal head dimension. Pelvimetry studies external and internal maternal pelvic diameters measured in a static position. Their evaluation was intended to correlate such variables to prevent the clinical consequences of labor dystocia and operative delivery [2]. 

The pubis is a critical reference point for the second stage of labor [3]. The pubic arch angle (PAA) is geometrically defined by the inferior aspect of the pubis symphysis, the ischio-pubic branches' arch, and the ischial tuberosities. Pelvimetry has established that the static width of the PAA and outlet's bi-tuberous diameter assessed in the position of the supine flexed legs is involved in the length of the second phase of labor and the occurrence of unplanned obstetrical intervention [3,4]. When evaluated with 2D and 3D sonography, the static pubic arch amplitude measured in the supine maternal 45-degrees flexed legs position efficiently predicts the risk of dystocia and, consequently, anal sphincter trauma and incontinence after delivery [3,5].

Relaxin and estrogen placental production increase ligament hydration and looseness, particularly in the pelvic area, for the dynamic processes of birth biomechanics and making room for the passage of the fetus, facilitated by maternal posture [2]. The mouldability/diastasis of the pubic symphysis could significantly influence the degrees of motion of the sacroiliac joint [6]. An insufficiency or excess in the hydration of the joint connective tissue can cause musculoskeletal pain or a higher risk of sore subluxation and dislocation [1]. The mobility of the pelvic and lumbar joints has been identified as a vital factor, as poor mobility is often associated with lower back pain, poor dynamic balance, and decreased performance [6,7]. Pelvic mobility refers to the ability of the pelvic structures to move and adjust during maternal shifting positions [2]. Pelvic mobility depends on the mobility of pelvic joints: hip joints, sacroiliac joints, sacrococcygeal junction, and pubic symphysis. Decreased pelvic mobility is related to lumbosacral pains during pregnancy [1,6,8]. Pelvic girdle pain during pregnancy is a prevalent concern among pregnant women, contributing to rising morbidity and disability rates, as well as increasing the likelihood of not-planned cesarean sections during labor [7]. Pelvic mobility is clinically and instrumentally measurable by the difference in the width of the bony distances in different postures [2,6,9].

Only a few studies have considered whether maternal posture modifies the dimension of external and internal pelvic diameters, the PAA included, and how soft tissues affect visceral mobility, influencing obstetric outcomes [2,10-15]. Gravity and posture interact during pregnancy; changes in body weight distribution and center of gravity, caused by gravity balance and postural alterations, can significantly impact pelvic joint mobility and several physiological systems [8]. Research for understanding, assessing, and supporting the dynamic interplay between the maternal pelvis and the fetal presentation is fundamental in providing quality care during pregnancy and childbirth and screening adverse outcomes for both the mother and the baby [2,4,7,10,14]. 

Maternal connective-fascial articular and visceral tissues modify during pregnancy, maintaining mobility to preserve the pelvic floor physiology during pregnancy, childbirth, and puerperium [8-10]. Research showed that the PAA increases amplitude during pregnancy, confirming time-dynamic pelvic tissue adjustment, elasticity, and mobility [5]. A biomechanics study with 3D computational reconstruction from ultrasound images revealed changes in the pubis rotation angle with maternal posture and a 1 mm increase in pubic symphysis width when transitioning from a supine to a bent-leg position [9]. Progressively, the maternal pelvis adapts the size of its diameters and maintains its mobility throughout pregnancy. It was radiologically and clinically demonstrated in changing positions [2,3,11-13].

Ultrasound is a safe and comfortable real-time clinical assessment method useful for biomechanics research in obstetrics [3,5,9,10]. The pubic symphysis tissues, the pubic arch angle postural amplitude, and the degree of changes (DoC) of the PAA width are essential topics in the biomechanics of childbirth. Their study will complete a lack in the clinical evaluation of the maternal pelvic space and diameters in obstetric pelvimetry [2,11-15]. The paper describes maternal postures and the 2D ultrasound adapted technique to estimate the PAA change in shifting positions concerning different bent-leg hip flexion degrees. It will be employed for future biomechanics and clinical research assessing pelvic tissue adaptation of the birth canal during pregnancy and labor to prevent labor dystocia and its clinical complications. Ongoing research aims to elucidate further the relationship between pelvic dimensions, fetal progression, and obstetric outcomes, contributing to safer, more effective childbirth practices.

## Technical report

The PAA can be measured in different maternal positions: the difference in width between positions will show the quality of tissue adaptability. The patient's positions concern the extension-flexion degree of the hips, which is the foremost effective of lumbar and pelvis mobilization [11-15]. Furthermore, maternal supine, kneeling, and standing postures are differently influenced by gravity, leading to possible different modifications of the amplitude of the subpubic angle. Previous reports on the pelvimetry evaluation in changing maternal positions showed the ability to evaluate the DoC of pelvic diameters and fetal head station in different maternal postures [2,11-17].

Supine positions

**Figure 1:**
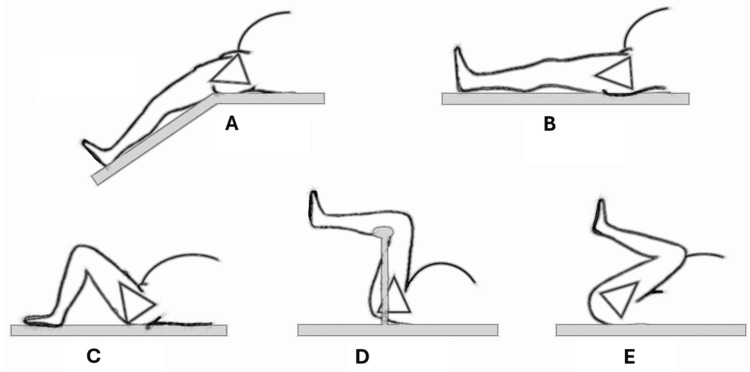
Supine leg positions for PAA measurements. The pelvic joints are mobilized by varying the degree of hip flexion-extension, influencing the pelvic space. A: Walcher position. B: Supine position (p1). C: 45° bent leg position (p2). D: 90° bent leg position. E: Hyperflexed leg position (p3). PAA: Pubic arch angle Image credit: Marco Siccardi.

To save the patient's compliance and convenience in the supine postures, the patient lies supine on the examination table with straight legs, like a standard supine position, without hip abduction or adduction. For preparing the examination, the patient flexes the legs to nearly 90 degrees, with feet in contact with the table and heels close to the ischium. The patient should press the feet against the table, lift the sacrum momentarily, and lower the pelvis back down. Different patient supine leg positions could be used for clinical studies: the hyperflexion position, as in the McRoberts maneuver; the hyperextended leg position, as in the Walcher's position; the 45-degree and 90-degree flex-leg position; the 90-degree flexion position with the legs sustained by stirrups (Figure 1) [13,14,16].

The sonographic convex probe is placed on the maternal anterior perineum. Sonographic measurement begins with the supine patient's legs flexed and then transitions to the straight-leg position by sliding the feet along the table surface. Maintaining the sonographic probe's contact with the perineum while the patient adjusts the leg position is preferable. In supine positions, maintaining consistent contact between the sonographic probe and the perineum is significantly more manageable compared to upright positions. The supine posture provides a stable and predictable anatomical orientation, reducing movement and variability. The stability of patients allows the operator to maintain a steady angle and consistent pressure on the probe without needing frequent adjustments. These factors make the supine position ideal for obtaining precise and reproducible ultrasound images with minimal difficulty in probe handling.

Kneeling and standing positions

**Figure 2:**
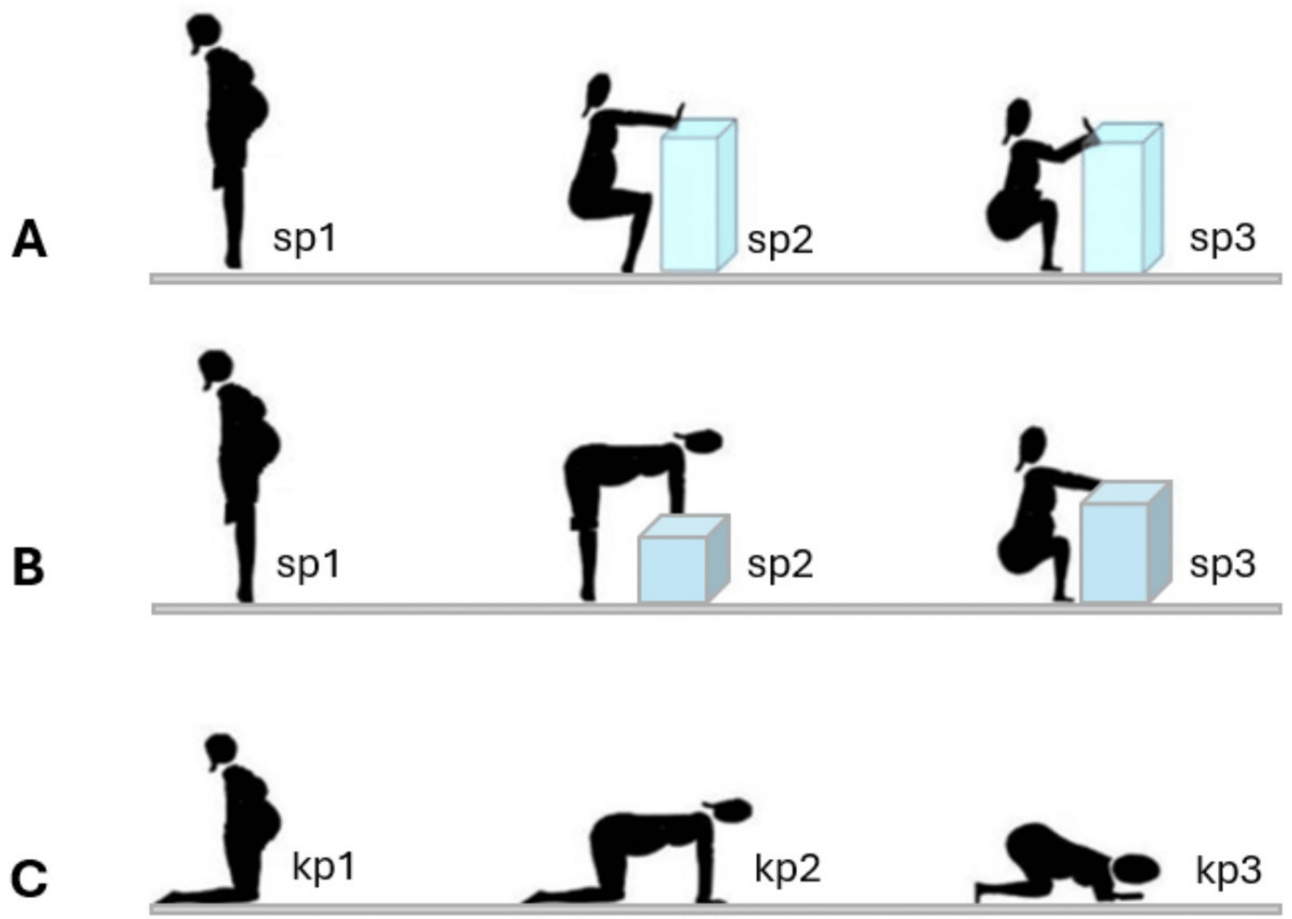
Standing and kneeling postures for pelvimetry evaluation. A, B: Standing positions. C: Kneeling positions. sp1: Standing erect position; sp2: standing 90° flexed leg position; sp3: hyperflexed leg position. kp1: kneeling erect position; sp2: "all-four" position; kp3: kneeling squat position. Image was modified from [17]. The authors granted permission to use and modify the image, published under the Creative Commons Attribution License CC-BY 4.0.

Women labor and give birth in positions where the sacrum is free to move, the flexible sacrum positions. The standing and kneeling postures used in obstetrics suitable for ultrasound measurement of the subpubic angle that vary with the degree of flexion of the lower limbs are the upright position, the "all-four" position, and the squat position. Our previous study analyzed potential postural changes for measuring pelvic diameters related to the birth canal. It evaluated the standing and kneeling postural procedures to estimate and quantify the dynamic degree of change in external pelvic diameters (Figure 2) [17].

Standing and kneeling positions represent the natural mechanics of childbirth, as they closely mimic the postures women adopt during labor and delivery. Unlike supine positions, standing allows the full engagement of gravitational forces. Maintaining sonographic probe contact in standing positions can be more challenging due to the patient's movement and the vertical orientation of the pelvis. As patients shift positions, their movements can cause the probe to lose contact, necessitating readjustment, particularly in the squat position. Frequent repositioning may lead to discomfort or fatigue for the patient, especially during prolonged procedures. Sonographers must develop expertise in dynamic probe handling, employ ergonomic supports, and maintain clear communication with patients to coordinate smooth and efficient positional transitions.

Sonographic technique

**Figure 3:**
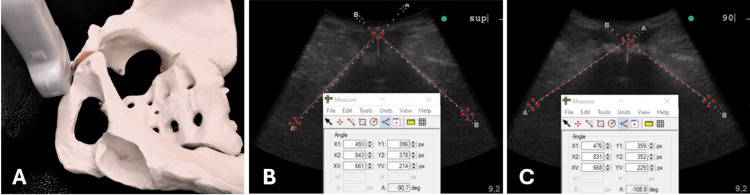
Perineal probe position and ultrasound PAA measurement. The optimal position of the probe on the perineum to visualize the ischio-pubic branches of the pubic arch angle is on the lower edge of the pubic symphysis at 45° tilt. The ultrasound PAA measurements of a multiparous non-pregnant supine patient demonstrate an amplitude difference of 18.2° between the hip extended and 90° hip flexed positions. A: The correct probe position and orientation on the perineum. B: Ultrasound image in the supine position (sup), PAA= 90.7°. C: Ultrasound image in the 90° flexed leg position (90), PAA= 108.9°. PAA: Pubic arch angle Image credit: Marco Siccardi.

The 2D ultrasound technique to acquire the proper scan of the image of the pubic arch angle is described in the literature [3]. After the patient is in the starting position, the ultrasound transducer, covered with a glove and ultrasound gel, is placed transversely near the lower edge of the pubic symphysis on the patient's anterior perineum and tilted at a 45° angle (Figure 3A). The tilting is likely done to optimize the imaging angle and obtain a clear view of the anatomical structures in the pelvic region, showing the pubic symphysis and the two symmetrical ischio-pubic rami. It is recommended that the sonographic probe remain in contact with the perineum during patient movements in changing positions to avoid patient discomfort and optimize procedure times. Lightly tilting the probe will find the correct image when the patient's posture is stable after the movement. The pubic angle is delineated by the inferior borders of the pubic rami that converge at the pubic symphysis. The PAA is measured by tracing bilaterally a line on the inferior pubic branches' medial edges, converging anteriorly on the middle of the pubic symphysis. It forms a triangle based on the ischial tuberosities posteriorly and the apex at the center of the symphysis (Figures 3B, 3C). If an optimal image is obtained, a single measurement of the PAA can be performed. Otherwise, the average of three measurements can be calculated.

## Discussion

The pioneering study of Borell and Fernstrom demonstrated a modification in pubis displacement and outlet diameters with maternal posture, indicating mobility in pubic symphysis and sacroiliac joint [14]. They showed that radiographic examinations in hip-flexed postures effectively diagnosed adequate pelvic space, enabling patients to proceed with labor and achieve uncomplicated deliveries. Their findings were confirmed by recent magnetic resonance (MR) research and computational reconstruction [9,10,15]. Increasing interest in recent years has produced clinical studies investigating pelvic postural biomechanics during pregnancy and childbirth. The PAA and bi-tuberous diameter are part of the pelvic outlet [3,4]. Ultrasound and MR research correlated the PAA with the pelvic inlet's anteroposterior diameter amplitude and the pelvic midplane's transverse diameter, confirming the pubis as a crucial fulcrum point in childbirth biomechanics [18]. Reitter et al. have shown under MR the modification of pelvic diameters and PAA with maternal position change in the degree of hip joint flexion [15]. The degree of hip joint flexion was associated with changes in the width of PAA and internal and external pelvic diameters [9,11,13,15].

The intrapartum angle of progression (AoP) is a perineal ultrasound evaluation to assess the progression of fetal descent during labor, providing important information about the fetal head's movement through the birth canal [16]. The angle is measured by aligning the longest axis of the pubic bone with the nearest bony part of the fetal head to the probe, representing the point of descent closest to the perineal plane. A wider AoP is considered favorable for the progression of labor: the fetal head can move further beyond the pubis. Zimerman et al. demonstrated that maternal hip flexion increases AoP values, with flexion >90° resulting in a maximal increase, as in McRoberts' maneuver (Figure 1D) [16]. Desseauve et al. determined with optoelectronic devices that hip flexion leads to upward displacement of the pubic symphysis, retroflection of the pelvic inlet plane, and decreased lumbar lordosis, getting closer to the theoretical perfect conditions during labor, characterized by an inlet plane perpendicular to the lumbar spine with no lumbar curve or promontory angle [12].

These findings are crucial for creating space for the descending fetus and highlight the potential for postural treatments in cases of obstructed labor [12,15,16]. Research by Siccardi et al. analyzed potential postural changes for measuring pelvic diameters related to the birth canal. They evaluated the standing and kneeling postural procedures to estimate and quantify the dynamic degree of change in those external pelvic diameters related to internal diameters: consistent improvement in pelvic spaces with postures was demonstrated, mainly in 90° flexed-leg postures [2,13,17]. The pelvic dynamics processes are inferred to be facilitated by the elasticity of the pubic symphysis and the soft tissues of the pelvic outlet. Data from all investigations support that changes in the pubic arch angle with maternal leg posture are significant, highlighting the importance of assessing tissue elasticity and adaptation to assist the physiology of labor [2-8,13,15]. 

The PAA was demonstrated to change its amplitude in different positions, more in the pregnancy period and the last trimester [5,15]. Our preliminary studies presented at the 2021 World Congress of Perinatal Medicine investigated measuring the pubic arch angle in two positions and correlating it to the small diameters of the external pelvimetry [19]. The exploratory cross-sectional pilot studies, involving 23 multiparous non-pregnant women with regular periods and without pelvic floor disorders, showed a straight-leg position (p1) value (mean±SD) of 95.7°± 9.8 and a bent-leg position (p2) value of 99.8°± 8.5 (Table 1). The mean p2-p1 difference was 4.1° (95% CI 1.45-6.3; p=0.004) [18]. The study's subjects were considerably higher than the minimum requirement for biomechanical studies [12]. The sonographic measurements of the PAA give a wider amplitude than the MR measurements reported in 50 pregnant and 50 non-pregnant women shifting positions (70°-74° vs. 75°-77°) [15]. However, the positional change differences (3°-5°) align between the two imaging methods, but 2D ultrasound is more accessible and commonly available in clinical practice.

**Table 1:** Pelvic parameter measurements in different leg positions. Data from 23 multiparous non-pregnant patients are shown in millimeters or degrees (°) as mean ± standard deviation. Data from [19]. PAA: Pubic arch angle; Td MSA: transverse diameter of the Michaelis area (the distance between the posterior superior iliac spines); H-Ld MSA: hemi-longitudinal diameter of the Michaelis area (the distance between the spinal process of the fifth lumbar vertebra and the second sacral segment). *: p-value from the permutation test for paired differences (https://www.statstodo.com/PairedDiff.php).

Pelvic parameter	Straight-leg position (p1)	90° flexed leg position (p2)	Hyper-flexed leg position (p3)	p-value*
PAA°	95.7° ± 9.8	99.8° ± 8.5		0.004
Base of Trillat's triangle	122.5 ± 7.1	110 ± 10		0.003
Bituberous diameter	77 ± 15.5	85.2 ± 15.6	97.2 ± 16	0.0005
Td MSA	125.8 ± 9	135 ± 7.8	136.9 ± 9.5	0.0005
h-Ld MSA	46.5 ± 6.8	62.2 ± 12.5	71.2 ± 13.1	0.0005

The PAA is part of obstetric external pelvimetry and is correlated to midplane transverse diameter [11,18]. The external pelvic diameters could be clinically measured in shifting positions by the external dynamic pelvimetry test [2,13]. Moreover, the communications reported the PAA and the base of the Trillat's triangle assessment (related to the pelvic midplane) in the supine position with legs extended and flexed to nearly 90° [19]. The transverse and hemi-longitudinal diameters (pelvic inlet) of the Michaelis sacral area (MSA) and the bi-tuberous diameter at the end of the ischiopubic branches (pelvic outlet) were measured from the kneeling erect position (p1) to the "all-fours" (p2) and kneeling squat (p3) positions [2,13,19]. The measuring instrument (Digital Distance Indicator, by Metrica SpA, Milano, Italy), firmly attached to the operator's fingers, allows the operator to maintain contact with the bony landmarks during position changes. The paired difference analysis showed a significant difference in the measurements between positions p1, p2, and p3 in the patients recruited, confirming the maternal pelvic adaptability to hip flexion degree postures (Table 1) [2,11,13]. 

Our introductory observation showed measurement differences between positions that were consistent with findings from larger samples [2,11,13-15]. The measurement of the PAA in the bent-leg position correlated with the longitudinal hemi-diameter of the Michaelis sacral area but not with the base of the Trillat triangle (the distance between the two inguinal creases measured on the upper edge of the pubis), as expected from biomechanics raw principles [2,13,19]. The second sacral segment and the tip of the fifth lumbar vertebra define the longitudinal hemi-diameter of Michaelis' area. Its measurement acts as a new modification of the Schober test on the flexion of the lumbosacral junction and sacral promontory angle [2,13,17]. Our results showed a linear correlation in the bent-leg position between the PAA and the longitudinal hemi-diameter of MSA. A clinical evaluation confirmed the reduction of the lumbar lordosis and sacral promontory angle with the hip flexion degree (as in the McRoberts and Gaskin maneuvers) obtained with optoelectronic devices [12,13]. The lack of correlation in postural dynamics DoC between PAA and the three transverse pelvic diameters suggests that the soft tissues of the pelvis may play an essential role in pelvic diameter measurements, as in the physiology of joint mobility and the process of childbirth [8,10,13]. 

Pelvic measurements must consider soft tissues and ligaments crucial for joint mobility and pelvic space during childbirth [8,13]. During the proposed procedure in the supine position, lifting the pelvis before taking the measurement relieves the fascia and connective tissues from the pressure of gravity, creating a neutral position. This adjustment helps stabilize the sacroiliac joints posteriorly and freely the pubic symphysis anteriorly. The ischiopubic branches can move more precisely when changing positions from leg flexion to extension. This procedure allows operators to verify the increased elasticity of the connective tissue in the pelvic joints during pregnancy, as occurs for pelvic viscera mobility [4,7,8,10]. When taking PAA measurements in flexible sacrum positions, lifting the pelvis from the table will be unnecessary. Changes in the internal and external pelvic diameters associated with positional shifts may provide insights into pelvic health during pregnancy, contributing to safer and more natural childbirth. During antenatal midwifery care, a midwife using a multi-professional approach can evaluate the PAA width and mobility with this method and suggest specific exercises to improve pubic and pelvic mobility before labor and prevent adverse obstetric outcomes [20].

Preventing intrapartum dystocia is an essential topic in obstetrics. No research has been performed comparing upright and supine positions to evaluate the influence of gravitational loading on pelvic diameters during pregnancy and childbirth. The 2D ultrasound imaging technique comfortably evaluates the PAA in horizontal and vertical leg-flexion positions. Zimerman et al. evaluated the modification during labor of the sonographic angle of progression of the fetal head's descent in the maternal supine posture with the hips in different flexed positions (Figure 1) [16]. Moreover, flexed-leg postures permit the mobility of the sacroiliac joint, pubic symphysis, and PAA, modify the internal and external pelvimetric diameters, and allow the maternal pelvis to disclose more room for fetal rotation and descent [2,8,11-15,19]. From the evidence mentioned above, a prospective clinical trial was registered, aiming to investigate the relationship between fetal progression ultrasound parameters evaluated during labor and maternal pelvis space clinically assessed during pregnancy and labor (https://ichgcp.net/clinical-trials-registry/NCT05718180). The dynamic external pelvimetry (DEP) test clinically evaluates the maternal pelvic space's postural mobility and adaptability [2,13,19]. The described sonographic procedure's flexibility allows for easy study of PAA elasticity and fetal AoP in supine, standing, and kneeling maternal positions. The DEP test and 2D ultrasound are potentially available in every clinical setting, even in resource-limited countries. Ultrasound is suitable for clinical research, offering greater accuracy and reproducibility than clinical examinations in diagnosing fetal head position and station and predicting labor arrest [3,16]. Abdominal ultrasound can determine head and spine positions, and transperineal ultrasound evaluates head station at lower levels. However, most studies measured parameters with women in a supine static position, overlooking the mobility, which is critical in accommodating fetal descent through dynamic changes in pelvic dimensions [3,5,18]. The registered study will assess variations in pelvic small diameters postural dimension and key ultrasound labor parameters in supine and kneeling squat positions, identifying cut-offs and their predictive value for a vaginal birth and risk of non-planned cesarean section in case of non-progressing labor. Understanding pelvic mobility and its impact on labor could improve obstetric clinical predictions and support safer deliveries.

While offering valuable insights into the natural biomechanics of childbirth, the standing position presents potential limitations to the sonographic procedure for measuring the PAA. One major challenge is maintaining consistent probe contact with the perineum due to the vertical orientation of the pelvis and the increased mobility of pelvic structures in this posture. Transitions between positions exacerbate this difficulty, as anatomical shifts and gravitational forces can displace the probe. Furthermore, while gravity in standing positions enhances the natural opening of the pelvic diameters, it also introduces variability in tissue tension and pelvic alignment, which may affect the reproducibility of measurements. Although critical for understanding labor dynamics, these gravitational effects complicate the standardization of sonographic assessments. Operators must adjust probe positioning and angle dynamically while managing the ergonomic challenges of maintaining contact in a less stable patient posture. Frequent repositioning may lead to discomfort or fatigue for the patient, especially during prolonged procedures. To address these challenges, operators must develop expertise in dynamic probe handling, employ ergonomic supports, and maintain clear communication with patients to coordinate smooth and efficient positional transitions. These factors underscore the need for refined techniques and equipment adaptations to optimize the accuracy and feasibility of sonographic measurements in these more physiologically representative positions.

## Conclusions

Postural pelvic joint mobility is essential for pregnancy and childbirth physiology. The elasticity of the soft tissues of the pubic symphysis and anterior perineum can be accurately assessed through the difference in measurement of the width of the PAA in maternal shifting leg positions. The postural degree of change of maternal pelvimetry measures and its impact related to the fetal head progression angle are worth studying as a screening test for obstructed delivery before and during labor. The proposed ultrasound-based approach for evaluating the PAA degree of change in various maternal positions offers a practical tool for research in labor management and predicting birth outcomes. Ongoing research aims to elucidate further the relationship between pelvic dimensions, fetal progression, and obstetric outcomes, contributing to safer, more effective childbirth practices. PAA amplitude and mobility, with the DEP test, could also be verified as a screening test for post-partum urinary incontinence and pelvic floor disorders.
